# Specific amino acid patterns define split specificities of HLA-B15 antigens enabling conversion from DNA-based typing to serological equivalents

**DOI:** 10.1007/s00251-020-01172-8

**Published:** 2020-06-20

**Authors:** Burcu Duygu, Benedict M. Matern, Lotte Wieten, Christina E.M. Voorter, Marcel G.J. Tilanus

**Affiliations:** 1 Transplantation Immunology, Tissue Typing Laboratory, Maastricht University Medical Center, P.O. Box 5800, 6202 AZ Maastricht, The Netherlands; 2 GROW, School for Oncology and Developmental Biology, Maastricht University, Maastricht, The Netherlands

**Keywords:** HLA-B*15 amino acid polymorphism, HLA B15 serological equivalents, HLA-B15 splits, LinkSēq™ HLA typing

## Abstract

The HLA-B15 typing by serological approaches defined the serological subgroups (or splits) B62, B63, B75, B76, B77 and B70 (B71 and B72). The scarcity of sera with specific anti-HLA antibodies makes the serological typing method difficult to discriminate a high variety of HLA antigens, especially between the B15 antigen subgroups. Advancements in DNA-based technologies have led to a switch from serological typing to high-resolution DNA typing methods. DNA sequencing techniques assign B15 specificity to all alleles in the HLA-B*15 allele group, without distinction of the serological split equivalents. However, the presence of antibodies in the patient defined as split B15 antigens urges the identification of HLA-B*15 allele subtypes of the donor, since the presence of donor-specific antibodies is an important contraindication for organ transplantation. Although the HLA dictionary comprises information regarding the serological subtypes of HLA alleles, there are currently 394 B15 antigens out of 516 in the IPD-IMGT/HLA database (3.38.0) without any assigned serological subtype. In this regard, we aimed to identify specific amino acid patterns for each B*15 serological split, in order to facilitate the assignment of B*15 alleles to serological equivalents after high-resolution molecular typing. As a result, serological specificities of 372/394 not yet assigned alleles could be predicted based on amino acid motifs. Furthermore, two new serological types were identified and added, B62-Bw4 and B71-Bw4.

**Electronic supplementary material:**

The online version of this article (10.1007/s00251-020-01172-8) contains supplementary material, which is available to authorized users.

## Introduction

Serological typing has been used for a long time to determine HLA typing of patients and donors. This method is based on complement-dependent cytotoxicity (CDC) test or microlymphocytotoxicity assay, which measures the reactivity of a panel of sera containing well-characterized anti-HLA antibodies (Terasaki and McClelland 1964). This technique has also been performed in the International Histocompatibility Workshops and resulted in the identification of different serological specificities of HLA genes including HLA-B15. In the HLA-B15 antigen group, the serological splits B62, B63, B75, B76, B77, and B70 (B71 and B72 split antigens) have been defined (Elsner et al. 2000; Hildebrand et al. 1994; Laundy et al. 1978; Lin et al. 1996; Steiner et al. 1997). However, the scarcity of sera with specific anti-HLA antibodies, in particular antibodies against serological splits or infrequent HLA antigens, makes it highly challenging to discriminate the high variety of serologically defined HLA antigens. Furthermore, the need for living cells to perform the CDC method creates additional challenges. The advancements in DNA-based technologies have led to a switch from serological to high-resolution DNA typing approaches to obtain a refined HLA typing of patients and donors (Erlich et al. 2001). A major advantage of the high-resolution full-length sequencing is the recognition of allele polymorphisms enabling distinction of epitopes. On the other hand, identification of serological specificities remains important for transplantation, especially for the determination of donor-specific antibodies (DSA) present in the patient’s serum. When an antibody against a B15 split antigen in the serum of the recipient is detected, the serological subtype of the B15 antigen present on the donor cells must be identified in order to determine whether this anti-B15 antibody is donor-specific. Because DSA can mediate and promote acute and chronic graft rejection, the presence of DSA is an important contraindication for solid organ transplantation (DeVos et al. 2011; Michielsen et al. 2019). In our current practice, high-resolution typing of HLA-B*15 is always performed for kidney patients and living donors. The serological subtype is determined using the HLA data dictionary (Holdsworth et al. 2009) from the IPD-IMGT/HLA database (Robinson et al. 2015). However, the number of alleles with assigned serological split types is limited in the database, and serological typing by CDC is not always possible to identify serological type due to the aforementioned limitations. Without knowing the B15 serological equivalent, the risk of rejection for patients having anti-B15-split HLA antibodies is present and therefore, all B*15 typed donors are considered contra-indicated for transplantation for this patient.

The expert assignment given by the HLA data dictionary (available as a searchable form in the IPD-IMGT/HLA database) has been generated with the data obtained from different sources, including the WHO Nomenclature Committee (Marsh et al. 2010), the International Cell Exchange (UCLA program), National Marrow Donor Program, and recent publications and individual laboratories (Holdsworth et al. 2009). There are 728 different B*15 alleles in the database (version 3.38.0), 37 of them are null or questionable alleles, and 516 are B15 antigens (based on 2-field allele assignment). Of these 516 antigens, 394 are without any serological assignment in the IPD-IMGT/HLA database (version 3.38.0). Neural network (NN) analysis increased the number of alleles assigned to serological split specificities (Maiers et al. 2003) but these results have already been included in the 2008 edition of the HLA data dictionary, as seen in the IPD-IMGT/HLA database website. In this study, we aim to provide a reliable, fast and straightforward method to predict serological specificities of HLA-B*15 alleles based on amino acid sequence patterns. For this purpose, we identified specific amino acid patterns for each B*15 serological subtype to predict the serological equivalents of B*15 alleles. In addition, we identified two new HLA-B*15 alleles by full-length allele-specific Sanger sequencing (Voorter et al. 2014); HLA-B*15:03:01:03 and HLA-B*15:16:01:03 and for both alleles, we predicted the serological specificity using our new approach and confirmed this by HLA class I serological typing.

## Materials and methods

### Dataset

#### HLA-B15 antigens

**Fig. 1:**
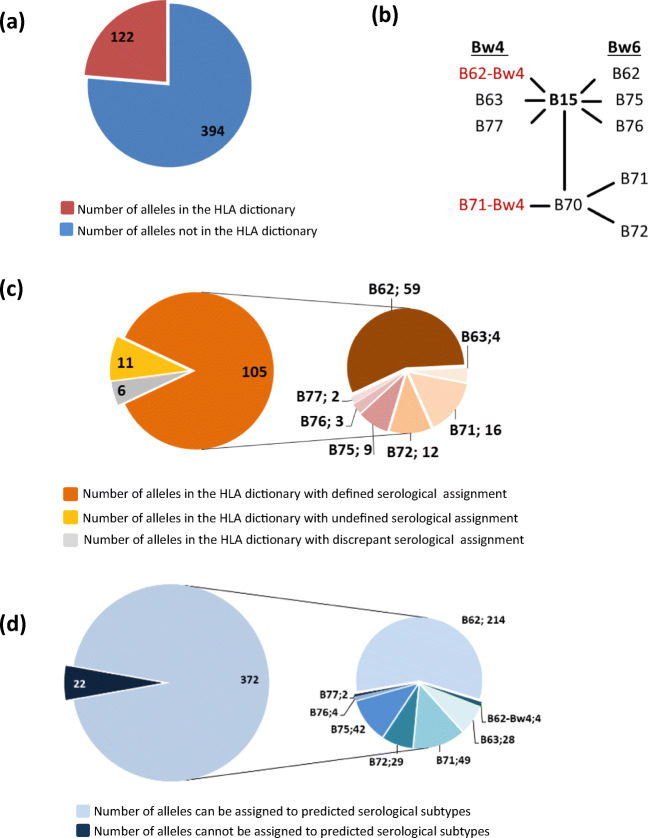
Overview of numbers of HLA-B15 antigens and B15 serological specificities reported in the IPD-IMGT/HLA database. **a** Number of HLA-B*15 alleles in IPD-IMGT/HLA database (release 3.38.0) present or absent in HLA dictionary. There are in total 516 HLA-B*15 alleles (number of alleles differing in 2nd field). **b** Serological subtypes of B15 antigen, separated according to Bw6 and Bw4 epitope presence. The new serological types B62 with Bw4 and B71 with Bw4 epitope are added in red. **c** Distribution of the 122 B15 alleles present in the dictionary according to serological assignment (for details, see Supplementary Table 1). **d** Distribution of the 372 B15 alleles absent from the dictionary according to predicted serological assignment (for details, see Supplementary Table 2)

HLA-B15 antigens included in the analysis have been obtained from the IPD-IMGT/HLA database (release 3.38.0) regarding each B*15 allele that differs in the second field as a different antigen and excluding the null and questionable alleles. In this way, we identified 516 different HLA-B15 antigens in the IPD-IMGT/HLA database (version 3.38.0) (Fig. 1a).

#### B15 antigens used for analysis (B*15 alleles in the dictionary)

The amino acid patterns of the different serological B15 split antigens were analysed, using only alleles with defined serological assignments in the HLA dictionary. The serological information from the expert assignment was used, and when this assignment is not conclusive, then the sources WHO and NN assignment were used. The original 2008 report of the HLA data dictionary included 123 HLA-B*15 alleles, but the previous B*95:30 is modified to B*15:27:02, resulting in 122 antigen-based HLA-B*15 alleles in total (Fig. 1a). Out of the 122, 105 have been assigned to a certain serological split antigen (supplementary Table 1) and these alleles have been used in this study to analyse the amino acid motif for each serological subtype (Figs. 1b and c and Table 1). For 6 alleles, the expert-assigned serological type is discrepant since the sources WHO assignment and neural network (NN) gave different serological assignments (Fig. 1c and Table 2). The remaining 11 alleles have an undefined expert assigned type (Fig. 1c and Table 3).

**Table 1:** Overview of 7 amino acid motifs characteristics for the B15 serological subtypes

	Amino acid positions						
	Exon 2						Exon 3
Serological types	24	45–46	63	65–67	70	77/80–83	166–167
B62	A	MA	E	QIS/QIF/QIC	N	S,NLRG	EW
B62-Bw4	A	MA	E	QIS	N	*N,IALR*	EW
B63	A	MA	E	*RNM*	**S**	*N,IALR*	EW
B71	*S*	*EE*	*N*	QIC/QIF/QIS	N	S,NLRG	EW
B71-Bw4	*S*	*EE*	*N*	QIC	N	*N,IALR*	EW
B72	*S*	*EE*	*E*	QIS	N	S,NLRG	EW
B75	A	MA	*N*	QIS/QIY/QIC	N	S,NLRG	EW
B76	A	MA	E	QIS	N	S,NLRG	*DG/ES*
B77	A	MA	*N*	QIS	N	*N,IALR*	EW

Amino acid motifs specific for each serological subtype are italicized

Numbers represent the amino acid positions

**Table 2:** Overview of B*15 alleles with discrepant serological assignment in the dictionary, with on the left side the characteristic amino acid motifs and on the right side the information from the dictionary and the serological assignment prediction based on the amino acid pattern

	Amino acid positions							The information in the HLA dictionary			
	Exon 2						Exon 3	Expert assigned	WHO Assigned	NN assigned	Based on aa pattern
Alleles	24	45–46	63	65–67	70	77/80–83	166–167				
B*15:08	A	MA	N	QIF	*N*	S, NLRG	EW	B75/62	B75(15)	B15	B75
B*15:15	A	MA	N	QIS	*N*	S, NLRG	EW	B75/62	B62(15)	B15	B75
B*15:23	S	EE	N	QIC	N	*N, IALR*	EW	B70/B5/blank	–	B70 B71	B71 Bw4
B*15:43	A	MA	E	QIS	N	*D, TLLR*	EW	B15	–	B62	B62 Bw4
B*15:87	A	MA	E	QIS	N	*S, IALR*	EW	B15		B15	B62 Bw4
B*15:115	S	EE	N	QIC	N	*S, TALR*	EW	B70	–	B70	B71 Bw4

Amino acid motifs specific for each serological subtype are italicized

Numbers indicate the amino acid positions. *WHO*, World Health Organization; *NN*, neural network; *aa*, amino acid

**Table 3:** Overview of B*15 alleles with undefined serological assignment in the dictionary, with on the left side the characteristic amino acid motifs and on the right side the information from the dictionary and the serological assignment prediction based on the amino acid pattern

	Amino acid positions							The information in the HLA dictionary			
	Exon 2						Exon 3	Expert assigned	WHO Assigned	NN assigned	Based on aa pattern
Alleles	24	45–46	63	65–67	70	77/80–83	166–167				
B*15:36	A	MA	E	QIS	N	N, TALR	EW	Undefined	–	B77	B62 Bw4
B*15:46	A	*KE*	E	QIS	N	S, NLRG	EW	Undefined	B72(70)	B15 B62	
B*15:52	S	EE	N	QIC	N	S, NLRG	EW	Undefined	B15	B71	B71
B*15:53	T	*KE*	E	QIS	N	S, NLRG	EW	Undefined	–	Not assigned	
B*15:62	S	EE	E	QIS	N	S, NLRG	EW	Undefined	–	B70 B72	B72
B*15:68	S	EE	E	QIS	N	S, NLRG	EW	Undefined	B35	B70 B72	B72
B*15:76	A	MA	N	QIY	*Q*	S, NLRG	EW	Undefined	–	B15	
B*15:86	A	MA	E	QIS	N	S, NLRG	EW	Undefined	–	B15 B62	B62
B*15:91	*S*	*EE*	E	QIS	N	S, NLRG	*ES*	Undefined	–	B70 B72 B12	
B*15:101	A	MA	E	QIY	*Q*	S, NLRG	ES	Undefined	–	B15 B46	
B*15:106	A	*KE*	E	QIS	N	S, NLRG	ES	Undefined	–	B15 B62	

Unique amino acid motifs that prevent prediction of serological assignment are italicized

Numbers indicate the amino acid positions

*WHO*, World Health Organization; *NN*, neural network; *aa*, amino acid

#### B*15 alleles used for prediction (B*15 alleles *not* in the dictionary)

394 B*15 alleles in the IPD-IMGT/HLA were not included in the HLA data dictionary (Fig. 1d). We used this set of alleles to predict the serological assignments by using the analysed amino acid patterns (Fig. 1d and Supplementary Table 2).

### Sequence-based typing

For ultrahigh-resolution typing, full-length allele-specific sequencing was performed by group-specific amplification and sequencing according to our previously published protocol (Voorter et al. 2014). In brief, allele group-specific amplification was performed with primers in 5′ and 3′ untranslated regions followed by Sanger sequencing using generic sequencing primers in both forward and reverse direction by means of cycle sequencing. The 3730 DNA-analyser was used for electrophoresis whereas analysis was performed with SeqPilot (JSI, Germany) and Lasergene (DNASTAR, Madison, Wisconsin) software, as previously described (Voorter et al. 2014).

### HLA class I serological typing

Heparinized blood was collected and lymphocytes were isolated by centrifugation on Ficoll-Hypaque. After counting the cells using pocH-100i (Sysmex) and adjusting to 4 × 10^6^ cells/ml, the serological typing was performed on this lymphocyte suspension using the standard NIH complement-dependent cytotoxicity (CDC) assay and a local set of sera. This local set of sera consists of 168 different sera, covering the HLA-A and HLA-B locus, and 12 negative and positive control sera. Sixteen of the sera were specific for HLA-B15 subtypes, 3 of them with monoclonal and 13 with polyclonal antibodies.

The standard NIH CDC assay in short: 1-ul cell suspension was added per well of typing trays, containing 1 μl of specific typing serum, and incubated 30 min at 20 °C. Complement activation was initiated by the addition of 5-μl rabbit complement (CEDARLANE®) and incubation at 20 °C for 60 min. After incubation with complement, FluoroQuench™ (Acridine orange (AO)/ ethidium bromide (EB) (One Lambda)) was used for staining, reading the trays by fluorescence microscopy after a 10-min incubation at room temperature in the dark. Trays were scored based on the percentage of dead and live cells and evaluated for serological typing assignment.

## Results

### Identification of specific amino acid motifs for each B15 serological subtype

HLA-B15 represents one of the largest broad antigen groups with different serological subtypes, containing B62, B63, B75, B76 and B77 specificities and is also associated with B71 and B72 (belonging to the B70 broad antigen) (Fig. 1b). The alignment of the 105 HLA-B*15 alleles with defined serological assignment in the HLA dictionary has led to the identification of in total 27 distinct patterns, which comprise amino acids that are different for at least two alleles. When an amino acid pattern alone or in combination with other amino acids is not specific for one subtype, these patterns are excluded, because they do not facilitate the designation of serological subtypes. In this way, 20 patterns have been excluded since they are not unique for a certain subtype (Supplementary Table 3). The remaining 7 amino acid motifs were identified as truly characteristic for the serological types (Table 1). These characteristic amino acid motifs were located at positions 24, 45–46, 63, 65–67, 70 and 166–167 (Table 1). In addition, amino acids at positions 77, 80, 81, 82 and 83 have already been described to characterize the Bw4 and Bw6 motifs (Muller et al. 1989). Bw4 specificity has been defined by the presence of N, D or S at residue 77 and IALR, TLLR or TALR amino acid motifs at residues 80–83(Lutz 2014). Since these motifs facilitate the distinction between serological subtypes, they were included in the analysis. B62 is the most common subtype and therefore used as a reference type. The difference in amino acid pattern compared with B62 is italicized in the table and these italicized motifs are used to determine each serological subtype. The amino acid pattern of ‘RNM and S’ at locations 65–67 and 70 together with Bw4 motif enabled prediction of B63 split antigen. Only the location 65–67 was already sufficient to specify B63 serological subtypes since ‘RNM’ is highly conserved among the B63 subgroup. Other serological subtypes all have one of the following combinations: QIC, QIF, QIS or QIY. For the B70 broad antigen, the amino acids S and EE at locations 24 and 45–46 are specific, whereas amino acid ‘N’ at location 63 specifies B71 and ‘E’ indicates B72 split antigens. B75 and B77 can be distinguished from B62 by the presence of an N at position 63. B77 carries, in addition to this N, the Bw4 motif, making this antigen in fact a B75-Bw4 type. Lastly, B76 antigen can be discriminated from B62 by the absence of the amino acids EW at locations 166 and 167. In the three known B76 antigens (B*15:12, 15:14 and 15:19), two different motifs are present, namely DG or ES. Since these amino acid patterns are quite distinct, it seems more logical that the B76 is defined as missing the EW motif, which fits with the serological finding that the B76 bearing cells react with less sera than the B62.

Furthermore, based on the 6 discrepant types (Table 2), we were able to identify two additional variants: B62-Bw4 which carries both B62 and Bw4 patterns (B*15:43 and B*15:87) and B71-Bw4, which bears both B71 and Bw4 patterns (B*15:23 and B*15:115). These variants are included in the list of serological types (Fig. 1b and Table 1). The remaining 2 discrepant HLA-B*15 alleles (B*15:08 and B*15:15) could be assigned to the serological subtype B75 according to their amino acid pattern (‘N’ at location 63 with Bw6 motif) (Tables 1 and 2).

The amino acid patterns of the 11 HLA-B15 antigens that were unassigned in the dictionary are shown in Table 3. Based on this pattern, 5 of them could be assigned to one of the 9 defined serological types. The remaining 6 alleles revealed unique amino acid patterns at designated locations, indicated in italic in Table 3, and therefore, the serological type could not be reliably predicted for these alleles.

### Verification of B15 serological splits of 2 new B*15 alleles

Two new HLA alleles, HLA-B*15:03:01:03 and HLA-B*15:16:01:03, were identified during routine high-resolution DNA typing of a kidney patient and a donor by full-length allele-specific sequencing method (Supplementary Fig. 1). The full-length sequences were confirmed by sequencing 2 different polymerase chain reaction products, from both individuals. The HLA-B*15:03:01:03 was most similar to HLA-B*15:03:01:02 with one nucleotide difference at position 1054 (G>T) in intron 3 while HLA-B*15:16:01:03 resembled HLA-B*15:16:01:01 with a single nucleotide change at position 119 (G>C) in intron 1 (Supplementary Fig. 1). In both cases, the new allele showed no amino acid differences with the most similar allele, since the single nucleotide changes were detected in the introns. The genomic sequences of these new alleles have been submitted to the EMBL Nucleotide Sequence Database (accession numbers LT618821 and LT898179 respectively) using a new allele submission tool called saddlebags (Matern et al. 2018) and to the IPD-IMGT/HLA database. The names HLA-B*15:03:01:03 and HLA-B*15:16:01:03 have been officially assigned by the World Health Organization (WHO) Nomenclature Committee (Marsh et al. 2010). Serological typing of these two samples was performed and confirmed the presence of B70 in the B*15:03:01:03 sample and B63 in the B*15:16:01:03 individual, as was already assigned by the experts for B*15:03 and B*15:16 respectively, as well as predicted by the amino acid composition. The B*15:03:01:03 is most probably B72 subtype, but this could not be confirmed by the CDC method due to lack of B71 and B72 specific sera in our laboratory.

### Prediction of serological specificities of B*15 alleles without defined serological types

Amino acid motifs were applied to the B*15 alleles that were not included in the HLA dictionary and were without assignment of serological subtype. Three hundred seventy-two alleles out of 394 could be predicted to a serological subtype by using the amino acid pattern shown in Table 1. The alleles and the prediction are indicated in Supplementary Table 2. The remaining 22 unassigned alleles revealed unique amino acid combinations at the determined amino acid motif positions that were not present in the serological assigned alleles (Table 4). In 20 of these cases, it concerns either a change of amino acid 24, not being A or S; a change of amino acid 45, not being E or M; or a change of the combination of these two motifs, not being A-MA or S-EE. The influence of these amino acid changes compared with known HLA-B15 subtypes on the serological reaction is unclear and could not be determined by serological typing due to the lack of viable cells with these B15 alleles.

**Table 4:** Overview of the 22 B*15 alleles with unique amino acid combinations preventing serological assignment prediction

	Amino acid positions						
	Exon 2						Exon 3
Alleles	24	45–46	63	65–67	70	77 80–83	166–167
B*15:143	A	*KE*	N	QIS	N	S, NLRG	EW
B*15:183	*T*	MA	E	QIS	N	S, NLRG	EW
B*15:202	A	*TA*	E	QIS	N	S, NLRG	EW
B*15:212	*T*	*KE*	E	QIS	N	S, NLRG	EW
B*15:239	A	*TE*	E	QIS	N	S, NLRG	EW
B*15:251	*S*	*MA*	E	QIS	N	S, NLRG	EW
B*15:259	*A*	*EE*	E	QIS	N	S, NLRG	EW
B*15:308	A	*TE*	N	QIS	N	S, NLRG	EW
B*15:336	*T*	MA	E	QIS	N	S, NLRG	EW
B*15:345	*T*	MA	N	QIS	N	S, NLRG	EW
B*15:376	S	*TE*	E	QIS	N	S, NLRG	EW
B*15:392	A	MA	*G*	QIS	N	S, NLRG	EW
B*15:429	*S*	*MA*	E	QIS	N	S, NLRG	EW
B*15:430	A	MA	E	QIS	N	*S, KLRG*	EW
B*15:434	S	*GE*	N	QIC	N	S, NLRG	EW
B*15:436	S	*GE*	N	QIC	N	S, NLRG	EW
B*15:504	*A*	*EE*	N	QIC	N	S, NLRG	EW
B*15:511	*T*	EE	E	QIS	N	S, NLRG	EW
B*15:525	A	*KE*	E	QIS	N	S, NLRG	EW
B*15:545	*S*	*MA*	E	QIC	N	S, NLRG	EW
B*15:553	*A*	*EE*	N	QIF	N	S, NLRG	EW
B*15:556	*S*	*MA*	E	QIS	N	S, NLRG	EW

Unique amino acid motifs that prevent prediction of serological assignment are italicized

Numbers indicate the amino acid positions

## Discussion

In this study, we provide a straightforward approach to predict serological splits of HLA-B*15 alleles based on amino acid polymorphisms. HLA-B*15 represents the largest broad antigen comprising 9 different serological splits. Currently, 516 HLA-B15 antigens are found in the IPD-IMGT/HLA database (release 3.38.0 (Robinson et al. 2015)), while no information is available regarding serological subtype of 394 alleles. Advancements in molecular techniques have led to a switch from serological typing to DNA typing of HLA alleles. Although DNA typing enables easy and specific allelic distinctions, it does not provide information about the corresponding serological type of HLA antigens by itself. Therefore, despite the rapid increase in the number of identified HLA alleles, information about their serological subtype remains limited. In addition, the scarcity of sera, especially with anti-HLA antibodies against split antigens, limits serological methods to determine serological splits. For instance, due to the unavailability of a B72 specific antiserum, the serological type of the new HLA-15 allele, HLA-B*15:03:01:03, could be only assigned as B70 by CDC serotyping. However, based on amino acid motifs identified in this study, we could now assign this new allele to the B72 serological subtype. Thus, our new approach facilitates the determination of serological subtype of HLA-B*15 alleles based on their DNA sequence.

In 2003, the neural network (NN) analysis has been developed by a machine learning model with the polypeptide sequences of HLA-A, HLA-B and HLA-DRB1 alleles alongside well-defined serological subtypes in order to predict the split assignments of 393 alleles (Maiers et al. 2003). Subsequently, this computational model was able to predict serological assignments for most alleles (95% HLA-A, 85% HLA-B, 96% HLA-DRB1). The information from this analysis has been included in the HLA Dictionary in 2008 (Holdsworth et al. 2009). However, this method is not generally available and demands expert skills to be executed. Therefore, our new approach based on the distinct and specific amino acid patterns of each B15 serological split provides an easy and practical solution. After evaluation of the serological subtypes of 105 alleles and determining the defining amino acid motifs, it was possible to predict not only the serological types of 6 alleles with conflicting information about their serological group in the HLA dictionary but also 5 out of 11 HLA-B*15 alleles which were undefined according to expert assignment and 372 out of 394 B*15 alleles that were not yet assigned. The remaining 22 alleles carry different amino acids than the identified motifs, warranting further analysis for definite serological assignments.

The number of antigens assigned to each serological split greatly varies. In total, B62 is the largest subtype since it is the serological equivalent of 274 different B*15 alleles (both in the dictionary and predicted by us). The second subtype is B71 with 66 alleles assigned to this split. Furthermore, different B*15 alleles have been assigned 43 to B72, 53 to B75, 32 to B63, 7 to B76 and 4 to B77 serological subtype. The new types B62-Bw4 and B71-Bw4 were assigned for 7 and 2 B*15 alleles. From the total of 516 B*15 alleles, we were able to assign 488 to a specific serological split, whereas 28 remain undetermined, because their amino acid patterns do not match with the identified ones. Since there is no empirical evidence regarding their reactivity against any serological group, it is currently not possible to reliably predict their serological subtype.

The serological subtype of HLA alleles is of particular interest in the setting of organ and stem cell transplantation, especially when the patients’ sera contain antibodies against a split antigen. Current techniques for antibody profiling of patient sera, such as Luminex bead assays, allow the identification of anti-HLA antibodies at the split level(Picascia et al. 2012). Since the presence of donor-specific antibodies (DSA) in the patient serum has been associated with graft failure, the identification of donor HLA type at the split level remains to be crucial for successful transplantation outcome (Michielsen et al. 2019). For this reason, Eurotransplant, a non-profit organ allocation organization of 8 European member countries, recommends transplantation centers to report HLA typing for both organ donors and patients at the serological split antigen level in order to obtain optimal organ allocation (ETRL Newsletter issue 9).

HLA typing of donor and patients for solid organ transplantation is at the moment generally performed at low resolution. In this paper, we used high-resolution full-length sequencing results to determine the crucial positions needed for serological subtyping of B15. With this analysis, we can now determine which method will obtain sufficient information to perform the serological subtyping of this antigen. Since the NGS methods with full-length sequencing have become rather cost-effective, these methods are ideal for patients and living donors, because there are less time constraints. For deceased donors, this is not possible yet, because even the fastest NGS method will still take more than 24 h. Therefore, we have also investigated whether the crucial amino acid positions can be determined with the faster typing method of real-time PCR using the LinkSēq™ technique. We have therefore checked the primer recognition sites, recognizing one or more of the crucial amino acids determining the B15 subtypes. All primer combinations that recognized a B15 subtype were used for the primary analysis to determine which combinations were crucial to make the difference between the different serological subtypes. These crucial primer combinations are indicated in Supplementary Table 4, together with the reactions of the different B15 subtypes. From this table, it is clear that each B15 subtype can be recognized by a unique pattern of positive and negative reactions. This positive/negative reaction pattern fits perfectly with the specific amino acid patterns identified in this study. Therefore, accurate prediction of B15 subtypes from a potential deceased donor is definitely possible, even during night shifts, revealing the possibility of exclusion based on the presence of antibodies against a certain B15 subtype for a potential recipient.

The presence of donor-specific antibodies is also important in the setting of stem cell transplantation (SCT) as it can influence donor engraftment and transplant outcome (Ciurea et al. 2018) and is especially important in the haploidentical transplantation setting, where one HLA haplotype is completely mismatched with the patient. For SCT, both patient and donor high-resolution HLA typing are performed in most centers, enabling conversion of HLA-B*15 alleles to serological split equivalents with our approach, to determine whether the antibodies present in the patient are indeed donor-specific.

In conclusion, we provide a straightforward practical approach to predict serological subtypes of HLA-B*15 alleles. This approach is useful for patients waiting for a stem cell or solid organ transplants that have antibodies against B15 subtypes, by predicting the donors’ B15 subtype and therewith circumventing donor-specific antibodies that have an impact on graft survival and acute and chronic rejection.

## Electronic supplementary material

ESM 1 (PDF 218 kb)

ESM 2 (DOCX 17 kb)

ESM 3 (DOCX 19 kb)

ESM 4 (DOCX 14 kb)

ESM 5 (DOCX 15 kb)

## Data Availability

The data that support the findings of this study are provided as a supplement to the manuscript. The amino acid sequences of HLA-B*15 alleles are derived from the IPD-IMGT/HLA database (Robinson et al. 2015) and two new HLA-B*15 allele sequences are available in the IPD-IMGT/HLA database (Robinson et al. 2015).
