# Supplementary material for: Specific amino acid patterns define split specificities of HLA-B15 antigens enabling conversion from DNA-based typing to serological equivalents
Source: Immunogenetics. 2020 Jun 20;72(6):339–46. doi: 10.1007/s00251-020-01172-8 (PMC7456404; doi:10.1007/s00251-020-01172-8)
Supplement: Supplementary file 1 — (PDF 218 kb) [file 251_2020_1172_MOESM1_ESM.pdf]

Supplementary Figure

```
(A)
gDNA                 1000       1010       1020       1030       1040       1050       1060       1070       1080       1090
B*15:03:01:02    GTACCAGG GGCAGTGGGG AGCCTTCCCC ATCTCCTATA GGTCGCCGGG GATGGCCTCC CACGAGAAGA GGAGGAAAAT GGGATCAGCG CTAGAATGTC
B*15:03:01:03    -------- ---------- ---------- ---------- ---------- ---------- ---T------ ---------- ---------- ----------

gDNA                 1100       1110       1120       1130       1140       1150       1160       1170       1180       1190
B*15:03:01:02  GCCCTCCCTT GAATGGAGAA TGGCATGAGT TTTCCTGAGT TTCCTCTGAG GGCCCCCTCT TCTCTCTAGG ACAATTAAGG GATGACGTCT CTGAGGAAAT
B*15:03:01:03  ---------- ---------- ---------- ---------- ---------- ---------- ---------- ---------- ---------- ----------

gDNA                 1200       1210       1220       1230       1240       1250       1260       1270       1280       1290
B*15:03:01:02  GGAGGGGAAG ACAGTCCCTA GGATAGTGAT CAGGGGTCCC CTTTGACCCC TGCAGCAGCC TTGGGAACCG TGACTTTTCC TCTCAGGCCT TGTTCTCTGC
B*15:03:01:03  ---------- ---------- ---------- ---------- ---------- ---------- ---------- ---------- ---------- ----------

gDNA                 1300       1310       1320       1330       1340       1350       1360       1370       1380       1390
B*15:03:01:02  CTCACACTCA GTGTGTTTGG GGCTCTGATT CCAGCACTTC TGAGTCACTT TACCTCCACT CAGATCAGGA GCAGAAGTCC CTGTTCCCCG CTCAGAGACT
B*15:03:01:03  ---------- ---------- ---------- ---------- ---------- ---------- ---------- ---------- ---------- ----------

gDNA                 1400       1410       1420       1430       1440       1450       1460       1470       1480       1490
B*15:03:01:02  CGAACTTTCC AATGAATAGG AGATTATCCC AGGTGCCTGC GTCCAGGCTG GTGTCTGGGT TCTGTGCCCC TTCCCTACCC CAGGTGTCCT GTCCATTCTC
B*15:03:01:03  ---------- ---------- ---------- ---------- ---------- ---------- ---------- ---------- ---------- ----------

gDNA                 1500       1510       1520       1530       1540       1550       1560
B*15:03:01:02  AGGCTGGTCA CATGGGTGGT CCTAGGGTGT CCCATGAGAG ATGCAAAGCG CCTGAATTTT CTGACTCTTC CCATCAG
B*15:03:01:03  ---------- ---------- ---------- ---------- ---------- ---------- ---------- -------


(B)
gDNA                   80         90        100        110        120        130        140        150        160        170
B*15:16:01:01     GTGAGTG CGGGTCGGGA GGGAAATGGC CTCTGTGGGG AGGAGCGAGG GGACCGCAGG CGGGGGCGCA GGACCCGGGG AGCCGCGCCG GGAGGAGGGT
B*15:16:01:03     ------- ---------- ---------- ---------- --------C- ---------- ---------- ---------- ---------- ----------

gDNA                       180        190        200
B*15:01:01:01     CGGGCGGGTC TCAGCCCCTC CTCGCCCCCA G
B*15:16:01:03     ---------- ---------- ---------- -
```
