# Supplementary material for: Specific amino acid patterns define split specificities of HLA-B15 antigens enabling conversion from DNA-based typing to serological equivalents
Source: Immunogenetics. 2020 Jun 20;72(6):339–46. doi: 10.1007/s00251-020-01172-8 (PMC7456404; doi:10.1007/s00251-020-01172-8)
Supplement: Supplementary file 2 — (DOCX 17 kb) [file 251_2020_1172_MOESM2_ESM.docx]

Supplementary Table 1: Overview of the HLA-B*15 alleles present in the dictionary with the serological assignment according to expert, WHO (World Health Organization) and NN (Neural Network). (Holdsworth et al. 2009)

| **HLA allele** | **Expert assigned** | **WHO assigned** | **NN assigned** |
| --- | --- | --- | --- |
| B*15:01 | B62 | B62(15) | Training - B15 B62 |
| B*15:02 | B75 | B75(15) | Training - B15 B75 |
| B*15:03 | B72 | B72(70) | Training - B70 B72 |
| B*15:04 | B62 | B62(15) | B62 |
| B*15:05 | B62 | B62(15) | B62 |
| B*15:06 | B62 | B62(15) | B62 |
| B*15:07 | B62 | B62(15) | Training - B15 B62 |
| B*15:08 | B75/B62 | B75(15) | B15 |
| B*15:09 | B70 | B70 | Training - B70 B71 |
| B*15:10 | B71 | B71(70) | Training - B70 B71 |
| B*15:11 | B75 | B75(15) | B15 |
| B*15:12 | B76 | B76(15) | Training - B15 B76 |
| B*15:13 | B77 | B77(15) | Training - B15 B77 |
| B*15:14 | B76 | B76(15) | B62 |
| B*15:15 | B75/62 | B62(15) | B15 |
| B*15:16 | B63 | B63(15) | Training - B15 B63 |
| B*15:17 | B63 | B63(15) | Training - B15 B63 |
| B*15:18 | B71 | B71(70) | Training - B70 B71 |
| B*15:19 | B76 | B76(15) | B76 |
| B*15:20 | B62 | B62(15) | B62 |
| B*15:21 | B75 | B75(15) | Training - B15 B75 |
| B*15:23 | B70/B5/blank | - | Not assigned |
| B*15:24 | B62 | B62(15) | B15 |
| B*15:25 | B62 | B62(15) | Training - B15 B62 |
| B*15:27 | B62 | B62(15) | Training - B15 B62 |
| B*15:28 | B62 | B62(15) | B62 |
| B*15:29 | B70 | B15 | B71 |
| B*15:30 | B62 | B62(15) | Training - B15 B62 |
| B*15:31 | B75 | B75(15) | B75 |
| B*15:32 | B62 | B62(15) | B62 |
| B*15:33 | B62 | B15 | B62 |
| B*15:34 | B62 | B62(15) | B62 |
| B*15:35 | B62 | B62(15) | Training - B15 B62 |
| B*15:36 | Undefined | - | B15 |
| B*15:37 | B70 | B70 | B71 |
| B*15:38 | B62 | - | B62 |
| B*15:39 | B62 | B62(15) | B62 |
| B*15:40 | B62/blank | - | B62 |
| B*15:42 | B15 | - | B62 |
| B*15:43 | B15 | - | B15 |
| B*15:44 | B75 | - | B75 |
| B*15:45 | B62 | B62(15) | B62 |
| B*15:46 | Undefined | B72(70) | Not assigned |
| B*15:47 | B70/blank | - | B72 |
| B*15:48 | B62 | B62(15) | B62 |
| B*15:49 | B72 | - | B72 |
| B*15:50 | B62 | - | B62 |
| B*15:51 | B70 | B70 | B71 |
| B*15:52 | Undefined | B15 | B71 |
| B*15:53 | Undefined | - | Not assigned |
| B*15:54 | B72 | - | B72 |
| B*15:55 | B15 | B15 | B75 |
| B*15:56 | B62 | - | B62 |
| B*15:57 | B62 | - | B62 |
| B*15:58 | B62 | B62(15) | B62 |
| B*15:60 | B62 | - | B62 |
| B*15:61 | B72 | - | B72 |
| B*15:62 | Undefined | - | Not assigned |
| B*15:63 | B62 | - | B62 |
| B*15:64 | B71 | - | B71 |
| B*15:65 | B62 | - | B62 |
| B*15:66 | B62 | - | B62 |
| B*15:67 | B63 | - | B63 |
| B*15:68 | Undefined | B35 | Not assigned |
| B*15:69 | B72 | - | B72 |
| B*15:70 | B62 | B62(15) | B62 |
| B*15:71 | B62 | B62(15) | B62 |
| B*15:72 | B71 | - | B71 |
| B*15:73 | B62 | B62(15) | B62 |
| B*15:74 | B72 | - | B72 |
| B*15:75 | B62 | - | B62 |
| B*15:76 | Undefined | - | Not assigned |
| B*15:77 | B62 | - | B62 |
| B*15:78 | B62 | B15 | B62 |
| B*15:80 | B70 | B70 | B71 |
| B*15:81 | B62 | - | B62 |
| B*15:82 | B62 | B62(15) | B62 |
| B*15:83 | B62 | - | B62 |
| B*15:84 | B62 | B62(15) | B62 |
| B*15:85 | B62 | - | B62 |
| B*15:86 | Undefined | - | Not assigned |
| B*15:87 | B15 | - | B15 |
| B*15:88 | B75 | - | B75 |
| B*15:89 | B77 | - | B77 |
| B*15:90 | B71 | - | B71 |
| B*15:91 | Undefined | - | Not assigned |
| B*15:92 | B62 | - | B62 |
| B*15:93 | B71 | B71(70) | B71 |
| B*15:95 | B63 | - | B63 |
| B*15:96 | B62 | B62(15) | B62 |
| B*15:97 | B62 | - | B62 |
| B*15:98 | B72 | - | B72 |
| B*15:99 | B71 | - | B71 |
| B*15:101 | Undefined | - | Not assigned |
| B*15:102 | B62 | B62(15) | B62 |
| B*15:103 | B70 | B70 | B72 |
| B*15:104 | B62 | - | B62 |
| B*15:105 | B62 | - | B62 |
| B*15:106 | Undefined | - | Not assigned |
| B*15:107 | B62 | B62(15) | B62 |
| B*15:108 | B71 | B71(70) | B71 |
| B*15:109 | B62 | - | B62 |
| B*15:110 | B62 | - | B62 |
| B*15:112 | B75 | B15 | B75 |
| B*15:113 | B62 | - | B62 |
| B*15:114 | B70 | B70 | B71 |
| B*15:115 | B70 | - | B70 |
| B*15:116 | B62 | - | B62 |
| B*15:117 | B62 | - | B62 |
| B*15:118 | B62 | - | B62 |
| B*15:119 | B71 | - | B71 |
| B*15:120 | B62 | - | B62 |
| B*15:121 | B75 | - | B75 |
| B*15:122 | B62 | - | B62 |
| B*15:123 | B72 | - | B72 |
| B*15:124 | B71 | - | B71 |
| B*15:125 | B62 | - | B62 |
| B*15:126 | B62 | - | B62 |
| B*15:127 | B72 | - | B72 |
| B*15:128 | B62 | - | B62 |
| B*15:129 | B62 | - | B62 |
| B*15:132 | B72 | - | B72 |
