# Supplementary material for: Specific amino acid patterns define split specificities of HLA-B15 antigens enabling conversion from DNA-based typing to serological equivalents
Source: Immunogenetics. 2020 Jun 20;72(6):339–46. doi: 10.1007/s00251-020-01172-8 (PMC7456404; doi:10.1007/s00251-020-01172-8)
Supplement: Supplementary file 3 — (DOCX 19 kb) [file 251_2020_1172_MOESM3_ESM.docx]

Supplementary Table 2: Overview of the 372 HLA-B*15 alleles with the predicted serological assignment according to the amino acid motifs.

| Alleles | Predicted assignment | Alleles | Predicted assignment | Alleles | Predicted assignment |
| --- | --- | --- | --- | --- | --- |
| B*15:131 | B72 | B*15:170 | B75 | B*15:213 | B75 |
| B*15:133 | B71 | B*15:171 | B62 | B*15:214 | B75 |
| B*15:134 | B71 | B*15:172 | B62 | B*15:215 | B75 |
| B*15:135 | B62 | B*15:173 | B72 | B*15:216 | B63 |
| B*15:136 | B62 | B*15:174 | B62 | B*15:217 | B62 |
| B*15:137 | B62 | B*15:175 | B62 | B*15:219 | B62 |
| B*15:138 | B62 | B*15:176 | B71 | B*15:220 | B72 |
| B*15:139 | B75 | B*15:177 | B63 | B*15:221 | B71 |
| B*15:140 | B62 | B*15:178 | B62 | B*15:222 | B63 |
| B*15:141 | B62 | B*15:179 | B62 | B*15:223 | B75 |
| B*15:142 | B62 | B*15:180 | B71 | B*15:224 | B62 |
| B*15:144 | B75 | B*15:184 | B62 | B*15:225 | B62 |
| B*15:145 | B62 | B*15:185 | B62 | B*15:227 | B62 |
| B*15:146 | B62 | B*15:186 | B71 | B*15:228 | B62 |
| B*15:147 | B62 | B*15:187 | B62 | B*15:229 | B71 |
| B*15:148 | B75 | B*15:188 | B62 | B*15:230 | B63 |
| B*15:150 | B62 | B*15:189 | B75 | B*15:231 | B62 |
| B*15:151 | B72 | B*15:191 | B75 | B*15:232 | B62 |
| B*15:152 | B62 | B*15:192 | B62 | B*15:233 | B62 |
| B*15:153 | B71 | B*15:193 | B62 | B*15:234 | B62 |
| B*15:154 | B62 | B*15:194 | B75 | B*15:235 | B72 |
| B*15:155 | B62 | B*15:195 | B62 | B*15:236 | B62 |
| B*15:156 | B72 | B*15:196 | B63 | B*15:237 | B62 |
| B*15:157 | B62-Bw4 | B*15:197 | B71 | B*15:238 | B71 |
| B*15:158 | B72 | B*15:198 | B71 | B*15:240 | B62 |
| B*15:159 | B62 | B*15:199 | B62 | B*15:241 | B62 |
| B*15:160 | B62 | B*15:200 | B71 | B*15:242 | B72 |
| B*15:161 | B71 | B*15:201 | B62 | B*15:243 | B72 |
| B*15:162 | B63 | B*15:203 | B62 | B*15:244 | B62 |
| B*15:163 | B62 | B*15:204 | B62 | B*15:247 | B62 |
| B*15:164 | B62 | B*15:205 | B62 | B*15:248 | B62 |
| B*15:165 | B62 | B*15:206 | B62 | B*15:249 | B62 |
| B*15:166 | B62 | B*15:207 | B62 | B*15:250 | B62 |
| B*15:167 | B62 | B*15:208 | B63 | B*15:252 | B71 |
| B*15:168 | B63 | B*15:210 | B72 | B*15:253 | B72 |
| B*15:169 | B62 | B*15:211 | B62 | B*15:254 | B63 |
| B*15:255 | B72 | B*15:299 | B62 | B*15:343 | B62 |
| B*15:256 | B62-Bw4 | B*15:300 | B62 | B*15:344 | B62 |
| B*15:257 | B62 | B*15:301 | B75 | B*15:346 | B62 |
| B*15:260 | B62 | B*15:303 | B75 | B*15:347 | B62 |
| B*15:261 | B62 | B*15:305 | B75 | B*15:348 | B62 |
| B*15:263 | B71 | B*15:306 | B71 | B*15:349 | B62 |
| B*15:264 | B62 | B*15:307 | B71 | B*15:350 | B75 |
| B*15:265 | B75 | B*15:309 | B62 | B*15:351 | B71 |
| B*15:266 | B72 | B*15:310 | B62 | B*15:352 | B62 |
| B*15:267 | B62 | B*15:311 | B71 | B*15:353 | B62 |
| B*15:268 | B63 | B*15:312 | B71 | B*15:354 | B71 |
| B*15:269 | B62 | B*15:313 | B71 | B*15:355 | B62 |
| B*15:270 | B76 | B*15:314 | B71 | B*15:356 | B63 |
| B*15:271 | B62 | B*15:315 | B62 | B*15:357 | B75 |
| B*15:273 | B63 | B*15:316 | B62 | B*15:358 | B75 |
| B*15:274 | B72 | B*15:317 | B62 | B*15:359 | B62 |
| B*15:275 | B71 | B*15:318 | B62 | B*15:360 | B62 |
| B*15:276 | B62 | B*15:319 | B75 | B*15:361 | B63 |
| B*15:277 | B62 | B*15:320 | B62 | B*15:362 | B63 |
| B*15:278 | B62 | B*15:322 | B62 | B*15:363 | B62 |
| B*15:279 | B62 | B*15:323 | B71 | B*15:364 | B62 |
| B*15:280 | B62 | B*15:324 | B62 | B*15:365 | B62 |
| B*15:281 | B72 | B*15:325 | B75 | B*15:366 | B62 |
| B*15:282 | B72 | B*15:326 | B62 | B*15:367 | B75 |
| B*15:283 | B75 | B*15:327 | B62 | B*15:368 | B62 |
| B*15:284 | B75 | B*15:328 | B75 | B*15:369 | B72 |
| B*15:285 | B62 | B*15:329 | B71 | B*15:370 | B62 |
| B*15:286 | B62 | B*15:330 | B75 | B*15:371 | B62 |
| B*15:287 | B62 | B*15:331 | B62 | B*15:372 | B62 |
| B*15:288 | B62 | B*15:332 | B62 | B*15:373 | B62 |
| B*15:289 | B62 | B*15:333 | B62 | B*15:374 | B75 |
| B*15:290 | B71 | B*15:334 | B62 | B*15:378 | B75 |
| B*15:291 | B75 | B*15:335 | B71 | B*15:379 | B72 |
| B*15:292 | B71 | B*15:337 | B71 | B*15:381 | B62 |
| B*15:293 | B71 | B*15:338 | B71 | B*15:382 | B71 |
| B*15:295 | B62 | B*15:339 | B62-Bw4 | B*15:383 | B62 |
| B*15:296 | B62 | B*15:340 | B62 | B*15:384 | B75 |
| B*15:297 | B75 | B*15:341 | B75 | B*15:385 | B62 |
| B*15:298 | B76 | B*15:342 | B62 | B*15:386 | B62 |
| B*15:387 | B62 | B*15:428 | B62 | B*15:473 | B62 |
| B*15:388 | B71 | B*15:431 | B62 | B*15:474 | B62 |
| B*15:389 | B72 | B*15:432 | B62 | B*15:475 | B72 |
| B*15:390 | B62 | B*15:433 | B72 | B*15:476 | B62 |
| B*15:391 | B62 | B*15:435 | B72 | B*15:477 | B62 |
| B*15:393 | B62 | B*15:437 | B75 | B*15:478 | B62 |
| B*15:394 | B75 | B*15:438 | B75 | B*15:479 | B62 |
| B*15:395 | B62 | B*15:439 | B71 | B*15:480 | B62 |
| B*15:396 | B63 | B*15:440 | B62 | B*15:481 | B62 |
| B*15:397 | B72 | B*15:441 | B62 | B*15:482 | B62 |
| B*15:398 | B62 | B*15:442 | B62-Bw4 | B*15:484 | B62 |
| B*15:399 | B75 | B*15:443 | B62 | B*15:485 | B62 |
| B*15:401 | B62 | B*15:444 | B72 | B*15:486 | B62 |
| B*15:402 | B62 | B*15:445 | B71 | B*15:488 | B62 |
| B*15:403 | B63 | B*15:446 | B63 | B*15:489 | B62 |
| B*15:404 | B75 | B*15:447 | B71 | B*15:490 | B62 |
| B*15:405 | B62 | B*15:448 | B72 | B*15:491 | B62 |
| B*15:406 | B62 | B*15:449 | B62 | B*15:492 | B62 |
| B*15:407 | B62 | B*15:450 | B62 | B*15:493 | B62 |
| B*15:408 | B63 | B*15:451 | B71 | B*15:494 | B62 |
| B*15:409 | B62 | B*15:452 | B62 | B*15:495 | B62 |
| B*15:410 | B62 | B*15:453 | B62 | B*15:497 | B62 |
| B*15:411 | B63 | B*15:455 | B71 | B*15:498 | B71 |
| B*15:412 | B62 | B*15:456 | B62 | B*15:499 | B62 |
| B*15:413 | B62 | B*15:457 | B62 | B*15:500 | B63 |
| B*15:414 | B71 | B*15:458 | B62 | B*15:501 | B62 |
| B*15:415 | B62 | B*15:459 | B62 | B*15:502 | B72 |
| B*15:416 | B62 | B*15:460 | B75 | B*15:503 | B71 |
| B*15:417 | B62 | B*15:461 | B62 | B*15:505 | B77 |
| B*15:418 | B77 | B*15:462 | B63 | B*15:506 | B71 |
| B*15:419 | B62 | B*15:464 | B62 | B*15:507 | B62 |
| B*15:420 | B75 | B*15:465 | B62 | B*15:508 | B75 |
| B*15:421 | B62 | B*15:466 | B62 | B*15:509 | B71 |
| B*15:422 | B62 | B*15:467 | B62 | B*15:510 | B62 |
| B*15:423 | B63 | B*15:468 | B62 | B*15:512 | B62 |
| B*15:424 | B63 | B*15:469 | B62 | B*15:513 | B75 |
| B*15:425 | B75 | B*15:470 | B62 | B*15:514 | B62 |
| B*15:426 | B71 | B*15:471 | B62 | B*15:515 | B62 |
| B*15:427 | B71 | B*15:472 | B62 | B*15:516 | B63 |
| B*15:517 | B72 | B*15:530 | B71 | B*15:541 | B62 |
| B*15:518 | B76 | B*15:531 | B62 | B*15:542 | B62 |
| B*15:519 | B62 | B*15:532 | B63 | B*15:543 | B62 |
| B*15:521 | B62 | B*15:533 | B62 | B*15:547 | B62 |
| B*15:522 | B62 | B*15:534 | B62 | B*15:548 | B71 |
| B*15:523 | B63 | B*15:535 | B62 | B*15:550 | B63 |
| B*15:524 | B62 | B*15:536 | B62 | B*15:551 | B76 |
| B*15:526 | B71 | B*15:537 | B75 | B*15:552 | B62 |
| B*15:527 | B72 | B*15:538 | B62 | B*15:554 | B62 |
| B*15:529 | B62 | B*15:539 | B72 | B*15:555 | B63 |
