# Supplementary material for: Specific amino acid patterns define split specificities of HLA-B15 antigens enabling conversion from DNA-based typing to serological equivalents
Source: Immunogenetics. 2020 Jun 20;72(6):339–46. doi: 10.1007/s00251-020-01172-8 (PMC7456404; doi:10.1007/s00251-020-01172-8)
Supplement: Supplementary file 4 — (DOCX 14 kb) [file 251_2020_1172_MOESM4_ESM.docx]

Supplementary table 3. Overview of amino acid patterns that are excluded as characteristic motifs to define serological subtypes.

| Amino acid positions | B62 | B62-Bw4 | B63 | B71 | B71-Bw4 | B72 | B75 | B76 | B77 |
| --- | --- | --- | --- | --- | --- | --- | --- | --- | --- |
| **4** | S | S | S/F | S | S | S | S | S | S |
| **11-12** | AM/SV/AV | AM | AM | AM | AM | AM | AM | AM | AM |
| **69*** | T | T | A | T | T | T/I | T | T | T |
| **71*** | T | T | A | T | T | T | T | T | T |
| **74** | Y/D | Y | Y | Y | Y | Y/D | Y | Y | Y |
| **94-95** | TL/TW/II | TL/II | TL/TW | TL/II | TL | TL | TL/II | TL | II |
| **97** | R/S/T/M/N | R | R | R | R | R | R/S | R | R |
| **99** | Y/F/S/C | Y | Y | Y | Y | Y | Y | Y | Y |
| **103** | V/L | V | V/L | V | V | V | V | V | V |
| **113** | H/Y | H/Y | H/Y | H | H | H | H/Y | H | Y |
| **114** | D/N | D | D/H | D/N | D | D | D | D | D |
| **116** | S/Y/L/F/D | S | S/D | S/Y | S | S | S | S | S |
| **131** | S/R | S | S | S/R | S | S | S | S | S |
| **143** | T/S | T | T | T/S | T | T | T | T | T |
| **147** | W/L | W | W | W/L | W | W | W | W | W |
| **152** | E/V/Q | E | E | E/V | E | E/V | E/V/T | E | E |
| **156** | W/L/D/R | W/L | L | W/L/D/R | L | W/L/R | L/W | W | L |
| **158** | A/T | A | A | A/V/T | A | A/T | A | A | A |
| **163** | L/E/T | L | L | L/E/T | L | L/E/T | L/T | L | L |
| **171** | Y/H | Y | Y | Y/H | Y | Y | Y | Y | Y |

* Although HLA-B*15:16 (B63), B*15:17 (B63), B*15:67 (B63), B*15:95 (B63) carry ‘A’ polymorphism at location 69 and 71,these locations have been excluded, because also the alleles B*15:76 and B*15:101, that are both undefined have the same ‘A’ polymorphism, making this pattern not characteristic for B63 subtype.
