# Supplementary material for: Specific amino acid patterns define split specificities of HLA-B15 antigens enabling conversion from DNA-based typing to serological equivalents
Source: Immunogenetics. 2020 Jun 20;72(6):339–46. doi: 10.1007/s00251-020-01172-8 (PMC7456404; doi:10.1007/s00251-020-01172-8)
Supplement: Supplementary file 5 — (DOCX 15 kb) [file 251_2020_1172_MOESM5_ESM.docx]

Supplementary Table 4: The reaction pattern of specific primer combinations from Linksēq™ assay determining HLA-B15 serological subtypes.

|  | Aminoacid sequences of primer combinations used in Linksēq™ | | | | | | | | | |
| --- | --- | --- | --- | --- | --- | --- | --- | --- | --- | --- |
| Serological types | 43P-RM-46A 60W-DRE-64T | 45M-APR-49A  62R-NTQ-66I | 41A-SPR-45E 60W-DR-63E | 11A-M-13S 23I-24S | 116S-AYD-120G  165V-DGL-169R (165V-ESL-169R) | 67S-KT-70N 80I-AL-83R | 69A-SAQ-73T 152E-AEQ-156L | 22F-ISV-26G 69T-NTQ-73T | 42S-PRE-46E 70N-TQT-74Y | 27Y-VDD-31T 80I-ALR-84Y |
| **B62** | + | - | - | - | - | - | - | - | - | - |
| **B62-Bw4** | + | - | - | - | - | - | - | - | - | + |
| **B63** | - | - | - | - | - | - | + | - | - | +/- |
| **B71** | - | - | - | + | - | - | - | + | + | - |
| **B71-Bw4** | - | - | - | + | - | - | - | + | + | - |
| **B72** | - | - | + | + /- | - | - | - | + | +/- | - |
| **B75** | - | + | - | - | - | - | - | - | - | - |
| **B76** | + | - | - | - | + | - | - | - | - | - |
| **B77** | - | + | - | - | - | +/- | - | - | - | + |

Primer sequences are given as aminoacid sequences. Numbers indicate start and end positions of primers on mature protein sequences of HLA-B15 antigens. Each primer set is used to amplify one specific location on the HLA gene. ‘+’represents positivity while ‘-‘ indicates negativity. ‘+/-‘ means that some alleles give positive reaction and some negative with corresponding primer sets. These primer combinations were taken from Linksēq™ assay (lot nummer K3691 and K3821).
